# Gain-of-Function Effects of N-Terminal *CEBPA* Mutations in Acute Myeloid Leukemia

**DOI:** 10.1002/bies.201900178

**Published:** 2019-12-23

**Authors:** Luisa Schmidt, Elizabeth Heyes, Florian Grebien

**Affiliations:** Institute for Medical Biochemistry University of Veterinary Medicine Vienna 1210, Austria

**Keywords:** acute myeloid leukemia, C/EBP*α* p30, *CEBPA* (CCAAT/enhancer-binding protein alpha), epigenetic landscape, transcription factor, truncated isoform

## Abstract

Mutations in the *CEBPA* gene are present in 10–15% of acute myeloid leukemia (AML) patients. The most frequent type of mutations leads to the expression of an N-terminally truncated variant of the transcription factor CCAAT/enhancer-binding protein alpha (C/EBP*α*), termed p30. While initial reports proposed that p30 represents a dominant-negative version of the wild-type C/EBP*α* protein, other studies show that p30 retains the capacity to actively regulate gene expression. Recent global transcriptomic and epigenomic analyses have advanced the understanding of the distinct roles of the p30 isoform in leukemogenesis. This review outlines direct and indirect effects of the C/EBP*α* p30 variant on oncogenic transformation of hematopoietic progenitor cells and discusses how studies of N-terminal *CEBPA* mutations in AML can be extrapolated to identify novel gain-of-function features in oncoproteins that arise from recurrent truncating mutations in transcription factors.

## 1 Introduction: C/EBP*α* Isoforms and Their Functions

The transcription factor CCAAT/enhancer-binding protein alpha (C/EBP*α*) is necessary for the development of several structures, including liver, airway epithelium, and adipose tissue.^[1,2]^ In the hematopoietic system, C/EBP*α* is important for stem and progenitor cell function and regulates the differentiation of myeloid cells.^[3]^ The intron-less *CEBPA* gene is located on chromosome 19.q13.1, and the usage of two translation initiation sites in the *CEBPA* mRNA can result in the expression of the full-length C/EBP*α* protein, p42 (42 kDa) and a shorter p30 isoform (30 kDa).^[4]^ The p30 isoform lacks two of three trans-activation elements (TE), but both isoforms share a third TE and the C-terminal basic-region leucine-zipper (bZIP) that is required for dimerization and DNA-binding.^[5,6]^ Both C/EBP*α* p30 and p42 can homo- or hetero-dimerize with other C/EBP family members and regulate the expression of genes that govern cell differentiation, growth, survival, metabolism, and inflammation.^[7]^ The p42/p30 ratio can influence cell fate: high p42 expression blocks cell proliferation and results in differentiation, whereas increased p30 levels are associated with an immature cell state and inhibition of terminal differentiation into adipocytes and neutrophil granulocytes.^[5,8,9]^ The expression ratio of the C/EBP*α* p42 versus p30 isoforms can be regulated by extracellular stimuli. Abundant nutrients and growth factors stimulate proliferation by enhancing the expression of an upstream open reading frame (uORF) in the *CEBPA* mRNA. Efficient uORF translation in nutrient-rich conditions promotes re-initiation of translation from the downstream start codon within the *CEBPA*-coding region, resulting in higher levels of C/EBP*α* p30.^[5,9]^

C/EBP*α* exerts several gene regulatory functions via its interaction with other transcription factors and epigenetic regulators. For instance, C/EBP*α* was shown to harbor pioneer factor activity and prime chromatin at myeloid-specific regions in cooperation with PU.1 and RUNX1.^[3,10]^ Furthermore, cooperative interactions between C/EBP*α* and GATA2 were shown to play a central role in eosinophil differentiation.^[1,2]^ The interplay of C/EBP*α* with histone acetyltransferases (such as CBP and p300) and the SWI/SNF chromatin remodeling complex is important for its gene regulatory functions, highlighting the critical role of C/EBP*α* within complex networks of proteins that are involved in epigenetic control of gene expression.^[11–13]^ In addition, C/EBP*α* p42 can directly interact with and stabilize p21 to inhibit cyclin dependent kinases, such as CDK4, thereby restraining cell cycle progression. Furthermore, direct suppression of E2F activity by interaction with p42 results in indirect inhibition of c-MYC expression and growth arrest.^[6,14–18]^

**Figure 1:**
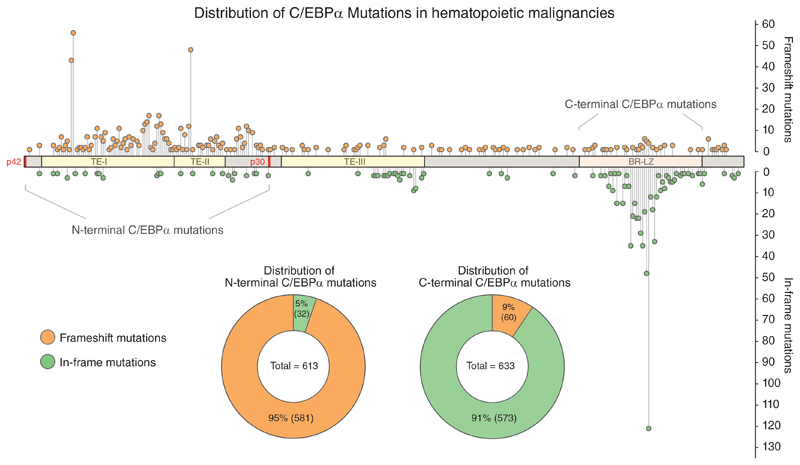
Overview of *CEBPA* mutations found in hematopoietic malignancies. Distribution and frequency of frame-shift (orange) and in-frame (green) mutations identified in the *CEBPA* gene in patients with hematopoietic malignancies. Data extracted from COSMIC database in 08/2019. TE, transactivation element; BR-LZ, basic-region leucine-zipper domain.

Besides C/EBP*α*, other transcription factors play important roles in the development and differentiation of hematopoietic cells, including RUNX1, PU.1, and GATA2.^[19]^ De-regulation of the transcription factor network and changes in protein levels can affect cell fate decisions and promote leukemogenesis. Indeed, mutated or de-regulated master transcription factors are regularly identified as oncogenic drivers in leukemia.^[19–27]^ Mutations in the *CEBPA* gene are found in 10–15% of de novo acute myeloid leukemia (AML) cases, predominantly in patients with normal karyotype acute myeloid leukemia (NK-AML).^[28–30]^ The mutations are not equally distributed across the *CEBPA* locus but cluster in two hotspots in different regions of the gene (Figure 1). N-terminal frameshift mutations result in the selective ablation of expression of the full-length C/EBP*α* p42 isoform. As the mutated region lies upstream of the internal translation initiation codon, they lead to exclusive expression of the shorter C/EBP*α* p30 variant. Alternatively, mutations in the C-terminal bZIP region disrupt the DNA-binding and/or dimerization ability of C/EBP*α*.^[1,5,6,28]^ By binding to co-factors, C-terminal mutated C/EBP*α* proteins can sequester these proteins away from chromatin, thereby functioning in a dominant-negative manner.^[5]^ AML patients frequently harbor an N-terminal frameshift mutation on one allele together with a C-terminal mutation on the other one, resulting in biallelic expression of different *CEBPA* mutants. Since 2016, AML with biallelic *CEBPA* mutations represents a separate disease entity in the World Health Organization (WHO) classification of myeloid neoplasms and acute leukemia, as it harbors few additional mutations, features a unique gene expression and immunophenotypic profile, and is associated with good prognosis.^[28,31–36]^ The p30 variant is often the only functional C/EBP*α* protein in leukemia cells with biallelic *CEBPA* mutations, but the molecular mechanisms underlying oncogenic transformation by N-terminal *CEBPA* mutations is unclear.^[1,28]^ In the following sections, we discuss recent advances in our understanding of C/EBP*α* p30-specific functions that might promote leukemogenesis.

## 2 C/EBP*α* p30-A Gain-of-Function Variant?

### 2.1 C/EBP*α* p30 Actively Changes Global Gene Expression Programs

The hypothesis that C/EBP*α* p30 possesses functions that are distinct from p42 already emerged over a decade ago, when p30 was found to have the ability to bind to gene promoters and repress gene expression independently of p42 in hepatoma cells.^[37]^ Since then, several groups have provided evidence that p30 is a functional C/EBP*α* variant that is capable of selectively altering gene expression programs. For instance, p30 was shown to up-regulate the expression of Ubc9, which leads to a decrease in the p42/p30 ratio via addition of small ubiquitin-like modifier proteins (sumoylation) and subsequent down-regulation of p42 in leukemia cells.^[38]^ The resulting block in myeloid differentiation could be reversed by siRNA-mediated knockdown of Ubc9 and restoration of p42 levels. Furthermore, the peptidyl-prolyl isomerase PIN1 was up-regulated upon p30 overexpression.^[39]^ PIN1 is highly expressed in many cancers including AML and is known to increase the stability of the proto-oncogene c-Jun, resulting in a differentiation block.^[40]^ C/EBP*α* p30 was also shown to bind to the promoter of *miR-181a* and directly up-regulate its expression, which sensitizes AML cells to chemotherapy.^[41]^ Along the same lines, Hughes et al. discovered opposing roles of the p42 versus p30 C/EBP*α* isoforms in the regulation of the long non-coding RNA *UCA1*. While both isoforms were capable of directly binding to the promoter of *UCA1*, only p30 was able to induce *UCA1*-expression. In turn, high levels of *UCA1* promote cell growth and proliferation by inhibiting the expression of the cell cycle regulator p27^kip^.^[42]^

### 2.2 C/EBP*α* p30 Chromatin Binding Directly Regulates Gene Expression

While all the above-mentioned studies focused on individual genes, a recent study by Jakobsen et al. investigated global p30-dependent gene expression changes. The authors performed comparative analyses of transcriptomic data derived from a mouse model of *CEBPA*-mutated AML and from samples of AML patients with biallelic *CEBPA* mutations. By comparing these C/EBP*α*-mutated leukemic cells with normal GMPs (granulocyte-macrophage progenitors), which represent their closest healthy counterparts, 102 genes were identified whose expression is de-regulated in human and mouse cells in response to C/EBP*α* mutations. Therefore, this gene set likely represents conserved effectors that might be functionally implicated in *CEBPA*-mutated AML. Only a small subset (20 genes) was up-regulated in C/EBP*α*-mutated cells, including *ARPP21*, *NT5E*, and *ITGAX*. Of these, CD73, the gene product of the *NT5E* gene, was also present at higher protein levels in cells with C/EBP*α* mutations. Two upstream enhancer regions were found to physically interact with the *Nt5e* promoter and strongly activate transcription, which was elegantly demonstrated by quantative analysis of chromosome conformation capture assays (3C-qPCR). Indeed, the C/EBP*α* p30 isoform was bound to an upstream enhancer of *Nt5e* and regulates CD73 expression levels. CD73 catalyzes the conversion of adenosine monophosphate to adenosine. As a result of up-regulated CD73 expression, elevated levels of adenosine stimulated proliferation and inhibited apoptosis in leukemia cells with C/EBP*α* mutations. In line with this, down-regulation of CD73 expression via clustered regularly interspaced short palindromic repeats interference (CRISPRi) (targeting the p30-bound enhancer) or RNA-interference significantly extended survival in a mouse model of *CEBPA*-mutated AML. Adenosine signaling requires members of the adenosine G-protein-coupled receptor family, which comprises four members: the A1 receptor, A2A receptor (A2AR), A2B receptor, and A3 receptor. Of these, only A2AR (encoded by the *ADORA2A* gene) was highly expressed in mouse and human C/EBP*α*-mutated cells.^[43]^ A2AR affects immune processes including pro-inflammatory cytokine secretion, macrophage-mediated phagocytosis, and C2 activation.^[44]^ In the context of *CEBPA*-mutated AML, adenosine signaling appears to function in a paracrine or autocrine manner through A2AR, as A2AR inhibition showed beneficial effects in a mouse model of *CEBPA*-mutated AML.

### 2.3 C/EBP*α* p30 Is Associated with Regulatory Regions across the Genome

In the same study, global chromatin association patterns of C/EBP*α* isoforms as well as epigenetic marks were assessed to characterize isoform-specific differences in p42- versus p30-expressing cells. Although both C/EBP*α* isoforms share the C-terminal DNA-binding domain, a subset of genomic regions (12.3%; 3815 of 30951 regions) was bound exclusively by either the p42 or the p30 isoform. Interestingly, most of these regions (63.7%; 2430 of 3815 regions) were occupied by the oncogenic p30 protein. While genomic regions with shared C/EBP*α* isoform binding as well as p42-specific binding sites harbored high-affinity C/EBP*α* consensus motifs, p30 localized to low-affinity C/EBP*α* binding sites. In addition, p30-occupied regions were enriched for transcription factor binding motifs for ERG, FLI1, and PU.1 proteins. In contrast, co-binding with the transcription factor HLF appears to be exclusive for p42, as only p42 binding sites harbored HLF consensus motifs and HLF expression was significantly down-regulated in p30-expressing cells. The findings of differential distribution of transcription factor binding motifs at isoform-exclusive genomic sites suggest that p42 and p30 can interact with different co-factors on chromatin. In addition to different distribution of transcription factor binding motifs, p42-bound regions were associated with lower histone 3 lysine 27 (H3K27) acetylation and lower histone 3 lysine 4 (H3K4) mono-methylation, while p30-bound regions as well as loci occupied by both p42 and p30 displayed high levels of these histone marks. As both H3K27 acetylation and H3K4 monomethylation are characteristics for enhancer regions, these results suggest that p30 preferably binds to primed enhancer regions, while p42 might be less dependent on the prior presence of chromatin marks that are associated with active transcription. This is consistent with data showing that C/EBP*α* can act as a pioneer factor in cooperation with other transcription factors, but it is not known if this feature is preserved in the truncated p30 variant of the protein.^[3,10]^ Based on these observations, it is proposed that higher levels of p30 protein as found in patients with *CEBPA* mutations enable p30 binding to low-affinity C/EBP*α* motifs at active and/or primed enhancers. In general, chromatin binding of either C/EBP*α* isoform was positively correlated with transcriptional activation, although the transcriptional activity of p30 is weaker than p42. This is in line with the observation that genes that are regulated by common p42/p30 binding to regulatory regions were more frequently down-regulated when p42 was missing. However, the expression of a different group of genes that is regulated by common p42/p30 binding was up-regulated in the absence of p42. Another recent publication also demonstrates opposing effects of C/EBP*α* isoforms on gene regulation. Chromatin binding of the transcription factor MYB, which is often de-regulated in leukemia at p30-specific sites was correlated with activated transcription, while the expression of genes in the proximity of regions bound by MYB together with p42 was predominantly repressed.^[45]^ These data point toward specific generegulatory functions of p30 that result in aberrant gene expression and oncogenic transformation.

### 2.4 C/EBP*α* p30 Preferentially Interacts with the MLL1 Protein Complex

The C/EBP*α* p30 variant can also exert specific functions through its interaction with epigenetic regulators. Affinity purification followed by mass spectrometry and co-immunoprecipitation studies found that the p30 isoform preferentially interacted with the MLL1 methyltransferase complex, which catalyzes H3K4 trimethylation.^[46,47]^ Consistently, there was a large overlap in global chromatin association of C/EBP*α* p30 and the MLL1 complex. These data indicate a cooperation of p30 with the MLL1 methyltransferase complex in the regulation of genes that are critical for the oncogenic effect of p30. CRISPR/Cas9-mediated mutagenesis revealed that p30-expressing cells are dependent on a functional MLL1 protein.^[47]^ Disruption of the MLL1 complex function by small-molecule-mediated inhibition of the MLL1-Menin or MLL1-WDR5 interaction resulted in impaired proliferation, induction of differentiation, and elevated levels of apoptosis of *CEBPA*-mutated cells.^[46,47]^

Taken together, these data demonstrate that the C/EBP*α* p30 protein represents a functional isoform of C/EBP*α* that can exert oncogenic effects through binding to enhancers and promoters and/or via preferential interaction with epigenetic modifiers and co-factors, which results in the specific regulation of p30-target genes.

## 3 Perspective

### 3.1 Truncating Transcription Factor Mutations in Leukemia-A Recurrent Theme

Beyond N-terminal *CEBPA* mutations in AML, other scenarios exist where a truncated transcription factor isoform retains the capacity of actively changing gene expression in cancer. Patients with Down syndrome have a high risk of developing a transient myeloproliferative disorder, which frequently develops into acute megakaryocytic leukemia.^[48]^ In this context, mutations in the transcription factor *GATA1* result in the expression of an N-terminally truncated protein that was termed GATA1s.^[49]^ Like p30, GATA1s retains the ability to bind DNA and can act as a transcriptional activator. Knockdown of GATA1 in a GATA1s-only expressing cell line resulted in de-regulation of genes associated with proliferation, differentiation, and cell death, some of which could be direct GATA1s targets. For instance, GATA1s can bind to GATA1-binding sites in the promoter region of the *IL1A* gene and regulate its protein levels.^[50]^ Additionally, the selective binding of GATA1s, but not GATA1, to regions corresponding to 140 genes implicated in diverse cellular pathways was recently demonstrated.^[51]^ Similar to the C/EBP*α* isoforms, the genomic regions that were bound by the truncated GATA1s isoform were associated with distinct transcription factor binding motifs, suggesting altered, isoform-specific interactions. Furthermore, the presence of GATA1s in erythroid cells was associated with dysregulated H3K27 methylation and altered chromatin accessibility. Thus, GATA1s could contribute to cell growth and survival by changing the epigenetic landscape to drive the expression of a subset of genes via differential co-factors.^[50]^

### 3.2 De-regulated Transcription Factor Activity Drives Oncogenic Transformation

De-regulated expression of transcription factors is recurrently observed in leukemia, highlighting the critical importance of balanced transcription factor expression for hematopoietic homeostasis. For instance, deletion of an upstream regulatory element causes down-regulation of PU.1 expression and leads to AML development.^[52]^ In contrast, RUNX1 levels are increased in many cancers, especially in AML.^[53]^ Interestingly, both up- and down-regulation of the transcription factor GATA2 can promote leukemogenesis.^[54–58]^ These findings exemplify the strong impact that transcription factor imbalance can have on proliferation, differentiation, and death of hematopoietic progenitor cells. Furthermore, they highlight the need to elucidate the molecular mechanisms by which mutated or de-regulated transcription factors contribute to leukemogenesis.

### 3.3 Interplay of Effects That Promote C/EBP*α* p30-Dependent Oncogenesis

Several recent studies have proposed mechanisms by which the oncogenic C/EBP*α* p30 isoform can interfere with normal transcriptional regulation. The identification of genomic regions that are exclusively bound by p30 indicates that this N-terminally truncated variant of C/EBP*α* can participate in active transcriptional regulation of distinct gene sets. Thus, the recent novel insights into the oncogenic mechanisms of C/EBP*α* p30 illustrate how a truncating mutation in a transcription factor can result in a functional isoform that has gained novel molecular functions to enable AML development. High levels of p30 expression and/or the absence of p42, which is observed in *CEBPA*-mutated AML, might allow binding of p30 to genomic regions that harbor low-affinity C/EBP*α* motifs, thereby changing global C/EBP*α*-dependent gene expression. As p30 is the only functional C/EBP*α* isoform present in the majority of AML with biallelic *CEBPA* mutations, genes that are aberrantly regulated by p30 are likely to include important oncogenic effectors. However, the p30-mediated activation of genes is only one of several aspects by which isoform imbalance in *CEBPA*-mutated AML could promote leukemogenesis. Loss of p42 leads to de-repression of genes as well as down-regulation of genes that are activated by p42. As the p30 isoform lacks two of three TEs, p30 exhibits reduced capacity to activate gene expression. The p30-induced up-regulation of specific gene sets could therefore result from altered patterns of co-factor binding and/or p30-specific interaction with other transcription factors, a concept that is supported by the finding that p42- versus p30-bound genomic regions exhibit different repertoires of transcription factor binding motifs. While HLF could be a p42-specific co-factor, factors such as ETS, ERG, and FLI1 show higher association with p30. In contrast, the known C/EBP*α* co-factor MYB might not distinguish between C/EBP*α* isoforms for chromatin binding, but exert differential gene regulatory effects depending on its association with either C/EBP*α* isoform. These results highlight the strong context-dependent influence of co-factor interactions on protein function. C/EBP*α*-specific co-factors can also be epigenetic regulators, such as the MLL1 histone methyltransferase complex. Thus, it is possible that the interaction of p30 with the epigenetic machinery is important for activation of transcription in cooperation with other transcription factors.

## 4 Conclusion

**Figure 2:**
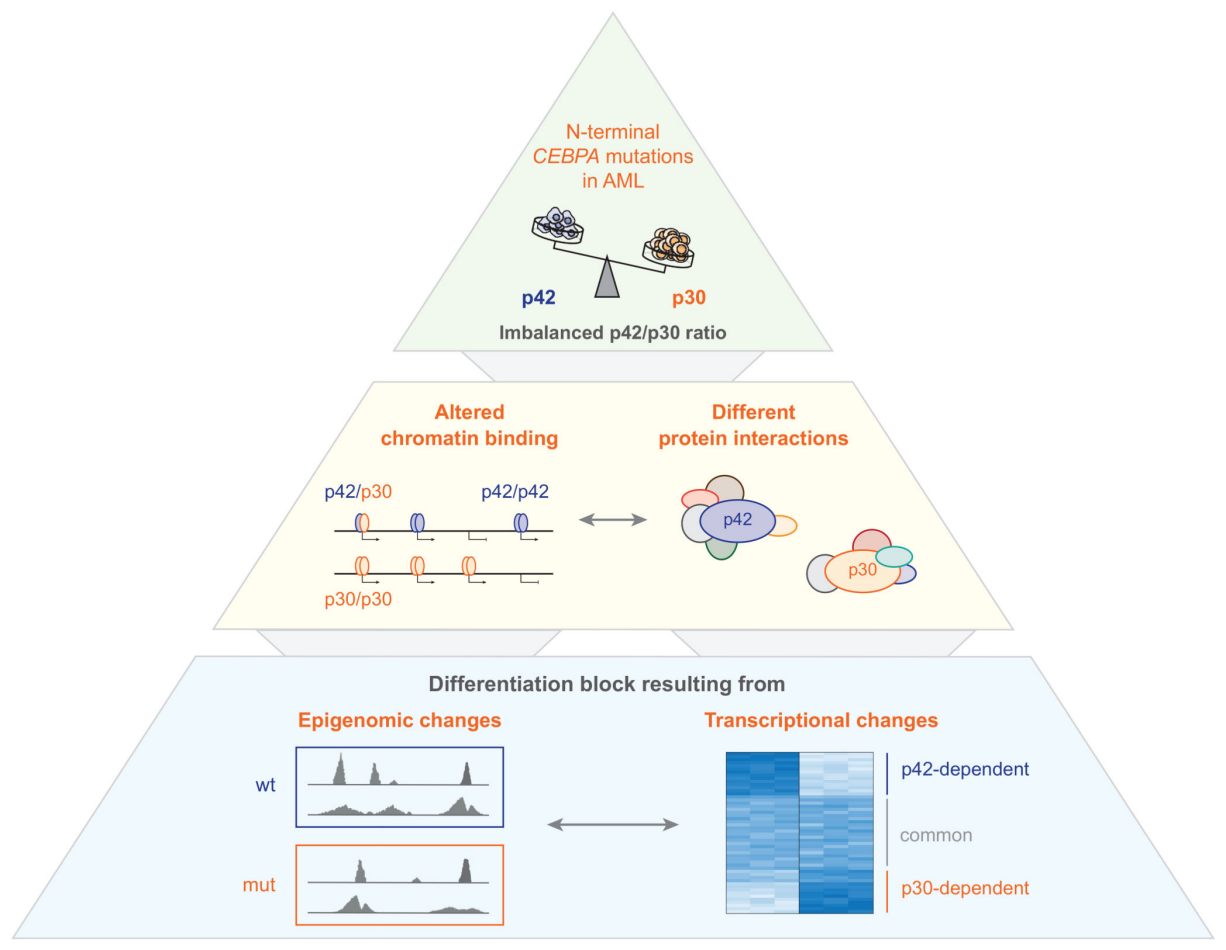
Summary of direct and indirect effects of C/EBP*α* p30 on oncogenic transformation. Schematic overview of the cause (green), effects (yellow), and consequences (blue) of a low p42/p30 ratio in N-terminal *CEBPA*-mutated AML resulting in differentiation block and leukemogenesis. wt, *CEBPA* wild-type; mut, N-terminal *CEBPA* mutation.

Taken together, recent evidence is compatible with a scenario where the truncated p30 isoform of C/EBP*α* is a gain-of-function allele that mediates oncogenic transformation via de-regulation of transcriptional programs. C/EBP*α* p30 displays altered chromatin binding properties that might depend on its interaction with other transcription factors and on different chromatin states. Specific interaction partners of p30 could promote binding of p30 to novel genomic regions to activate gene expression. The resulting global epigenomic and transcriptional changes that lead to oncogenic transformation demonstrate how aberrant expression of truncated yet functional isoforms of transcription factors can contribute to leukemogenesis (Figure 2).

## References

[1] Koschmieder S, Halmos B, Levantini E, Tenen DG (2009). J Clin Oncol.

[2] Nerlov C (2007). Trends Cell Biol.

[3] Avellino R, Delwel R (2017). Blood.

[4] Lin F-T, MacDougald OA, Diehl AM, Lane MD (1993). Proc Natl Acad Sci USA.

[5] Nerlov C (2004). Nat Rev Cancer.

[6] Leroy H, Roumier C, Huyghe P, Biggio V, Fenaux P, Preudhomme C (2005). Leukemia.

[7] Keeshan K, Santilli G, Corradini F, Perrotti D, Calabretta B (2003). Blood.

[8] Koschmieder S, D’Alò F, Radomska H, Schöneich C, Chang JS, Konopleva M, Kobayashi S, Levantini E, Suh N, Di Ruscio A, Vosco MT (2007). Blood.

[9] Calkhoven CF, Müller C, Leutz A (2000). Genes Dev.

[10] Hasemann MS, Lauridsen FKB, Waage J, Jakobsen JS, Frank A-K, Schuster MB, Rapin N, Bagger FO, Hoppe PS, Schroeder T, Porse BT (2014). PLoS Genet.

[11] Nerlov C, Ziff EB (1995). EMBO J.

[12] Pedersen TÅ, Kowenz-Leutz E, Leutz A, Nerlov C (2001). Genes Dev.

[13] Nerlov C, Ziff EB (1994). Genes Dev.

[14] Slomiany BA, D’Arigo KL, Kelly MM, Kurtz DT (2000). Mol Cell Biol.

[15] D’Alo’ F, Johansen LM, Nelson EA, Radomska HS, Evans EK, Zhang P, Nerlov C, Tenen DG (2003). Blood.

[16] Wang Q-F, Cleaves R, Kummalue T, Nerlov C, Friedman AD (2003). Oncogene.

[17] Johansen LM, Iwama A, Lodie TA, Felsher DW, Golub TR, Daniel G, Sasaki K, Tenen DG (2001). Mol Cell Biol.

[18] Timchenko NA, Wilde M, Nakanishi M, Smith JR, Darlington GJ (1996). Genes Dev.

[19] Imperato MR, Cauchy P, Obier N, Bonifer C (2015). Int J Hematol.

[20] Papaemmanuil E, Gerstung M, Bullinger L, Gaidzik VI, Paschka P, Roberts ND, Potter NE, Heuser M, Thol F, Bolli N, Gundem G (2016). N Engl J Med.

[21] Loke J, Assi SA, Imperato MR, Ptasinska A, Cauchy P, Grabovska Y, Soria NM, Raghavan M, Delwel HR, Cockerill PN, Heidenreich O (2017). Cell Rep.

[22] Osato M, Asou N, Abdalla E, Hoshino K, Yamasaki H, Okubo T, Suzushima H, Takatsuki K, Kanno T, Shigesada K, Ito Y (1999). Blood.

[23] Langabeer SE, Gale RE, Rollinson SJ, Morgan GJ, Linch DC (2002). Genes, Chromosomes Cancer.

[24] Vangala RK, Heiss-Neumann MS, Rangatia JS, Singh SM, Schoch C, Tenen DG, Hiddemann W, Behre G (2003). Blood.

[25] Mueller BU (2002). Blood.

[26] Mizuki M, Schwable J, Steur C, Choudhary C, Agrawal S, Sargin B, Steffen B, Matsumura I, Kanakura Y, Bohmer FD, Müller-Tidow C (2003). Blood.

[27] Tenen DG (2003). Nat Rev Cancer.

[28] Fasan A, Haferlach C, Alpermann T, Jeromin S, Grossmann V, Eder C, Weissmann S, Dicker F, Kohlmann A, Schindela S, Kern W (2014). Leukemia.

[29] Ahn J-S, Kim H-J, Kim Y-K, Lee S-S, Ahn S-Y, Jung S-H, Yang D-H, Lee J-J, Park HJ, Lee J-Y, Choi SH (2018). Oncotarget.

[30] Zhang Y, Wang F, Chen X, Liu W, Fang J, Wang M, Teng W, Cao P, Liu H (2018). Front Med.

[31] Konstandin NP, Pastore F, Herold T, Dufour A, Rothenberg-Thurley M, Hinrichsen T, Ksienzyk B, Tschuri S, Schneider S, Hoster E, Berdel WE (2018). Blood Adv.

[32] Arber DA, Orazi A, Hasserjian R, Borowitz MJ, Le Beau MM, Bloomfield CD, Cazzola M, Vardiman JW (2016). Blood.

[33] Pabst T, Eyholzer M, Fos J, Mueller BU (2009). Br J Cancer.

[34] Wouters BJ, Löwenberg B, Erpelinck-Verschueren CAJ, van Putten WLJ, Valk PJM, Delwel R (2009). Blood.

[35] Mannelli F, Ponziani V, Bencini S, Bonetti MI, Benelli M, Cutini I, Gianfaldoni G, Scappini B, Pancani F, Piccini M, Rondelli T (2017). Haematologica.

[36] Lin L-I, Chen C-Y, Lin D-T, Tsay W, Tang J-L, Yeh Y-C, Shen H-L, Su F-H, Yao M, Huang S-Y, Tien H-F (2005). Clin Cancer Res.

[37] Wang C, Chen X, Wang Y, Gong J, Hu G (2007). Cell Res.

[38] Geletu M, Balkhi MY, Peer Zada AA, Christopeit M, Pulikkan JA, Trivedi AK, Tenen DG, Behre G (2007). Blood.

[39] Pulikkan JA, Dengler V, Peer Zada AA, Kawasaki A, Geletu M, Pasalic Z, Bohlander SK, Ryo A, Tenen DG, Behre G (2010). Leukemia.

[40] Bao L, Kimzey A, Sauter G, Sowadski JM, Lu KP, Wang D-G (2004). Am J Pathol.

[41] Hickey CJ, Schwind S, Radomska HS, Dorrance AM, Santhanam R, Mishra A, Wu Y-Z, Alachkar H, Maharry K, Nicolet D, Mrózek K (2013). Blood.

[42] Hughes JM, Legnini I, Salvatori B, Masciarelli S, Marchioni M, Fazi F, Morlando M, Bozzoni I, Fatica A (2015). Oncotarget.

[43] Jakobsen JS, Laursen LG, Schuster MB, Pundhir S, Schoof E, Ge Y, D’Altri T, Vitting-Seerup K, Rapin N, Gentil C, Jendholm J (2019). Sci Adv.

[44] Gessi S, Merighi S, Sacchetto V, Simioni C, Borea PA (2011). Biochim Biophys Acta, Biomembr.

[45] Volpe G, Cauchy P, Walton DS, Ward C, Blakemore D, Bayley R, Clarke ML, Schmidt L, Nerlov C, Garcia P, Dumon S (2019). Life Sci Alliance.

[46] Grebien F, Vedadi M, Getlik M, Giambruno R, Grover A, Avellino R, Skucha A, Vittori S, Kuznetsova E, Smil D, Barsyte-Lovejoy D (2015). Nat Chem Biol.

[47] Schmidt L, Heyes E, Scheiblecker L, Eder T, Volpe G, Frampton J, Nerlov C, Valent P, Grembecka J, Grebien F (2019). Leukemia.

[48] Crispino JD (2005). Pediatr Blood Cancer.

[49] Wechsler J, Greene M, McDevitt MA, Anastasi J, Karp JE, Le Beau MM, Crispino JD (2002). Nat Genet.

[50] Xavier AC, Edwards H, Dombkowski AA, Balci TB, Berman JN, Dellaire G, Xie C, Buck SA, Matherly LH, Ge Y, Taub JW (2011). PLoS One.

[51] Ling T, Birger Y, Stankiewicz MJ, Ben-Haim N, Kalisky T, Rein A, Kugler E, Chen W, Fu C, Zhang K, Patel H (2019). Blood.

[52] Rosenbauer F, Wagner K, Kutok JL, Iwasaki H, Le Beau MM, Okuno Y, Akashi K, Fiering S, Tenen DG (2004). Nat Genet.

[53] Sun C-C, Li S-J, Chen Z-L, Li G, Zhang Q, Li D-J (2019). Mol Ther–Oncolytics.

[54] Zhang S-J, Ma L-Y, Huang Q-H, Li G, Gu B-W, Gao X-D, Shi J-Y, Wang Y-Y, Gao L, Cai X, Ren RB (2008). Proc Natl Acad Sci USA.

[55] Vicente C, Conchillo A, García-Sánchez MA, Odero MD (2012). Crit Rev Oncol/Hematol.

[56] Luesink M, Hollink IHIM, Van Der Velden VHJ, Knops RHJN, Boezeman JBM, Haas D, Trka J, Baruchel A, Reinhardt D, Van Der Reijden BA, van den Heuvel-Eibrink MM (2017). Blood.

[57] Chong C-E, Venugopal P, Stokes PH, Lee YK, Brautigan PJ, Yeung DTO, Babic M, Engler GA, Lane SW, Klingler-Hoffmann M, Matthews JM (2018). Leukemia.

[58] Nandakumar SK, Johnson K, Throm SL, Pestina TI, Neale G, Persons DA (2015). Exp Hematol.

